# Reminiscences and Experiences of an Old Physician

**Published:** 1899-03

**Authors:** G. G. Roy

**Affiliations:** Atlanta, Ga.


### REMINISCENCES AND EXPERIENCES OF AN OLD PHYSICIAN.

BY G. G. ROY, M.D.,

ATLANTA, GA.

In my last communication to THE JOURNAL, I wrote of "Typhoid Fever of Fifty Years Ago," and gave a history of my own case as learned from my father, Dr. A. G. D. Roy, and my old colored nurse, who had nursed me as an infant, and who kept faithful vigils over me during this sickness.

I propose now to speak of my first case of typhoid fever, after I hung out my shingle as a full-fledged doctor. As I have before mentioned in an article to THE JOURNAL, I began active practice with my father, and had the benefit of his experience and advice; but in my first case of fever, which I am now reporting—it seems that he forsook me, as well as Dr. Wm. Smith, another old and excellent physician, in the neighborhood. I asked them both to help me, but they said from all they could learn from me and others, the patient was bound to die and they did not feel justified in putting the family to additional and unnecessary expense, or it may have been; as the sequel will show, she had refused to have either of them. The subject was Mrs. Elizabeth S., wife of a Methodist preacher and school-teacher.

She had for several days the prodromata of a serious spell of illness. Her husband had begged her to let him send for my father or Dr. Smith. This she persistently refused to do. She said to him: "William, I am going to be 'mighty sick,' and I want you to send for Dr. Gus,"—my distinguishing title from my father, who was Dr. Roy.

This he declined to do for several days, telling her, "Elizabeth, Dr. Roy and Dr. Smith have practiced upon you since you were a child, and it seems very strange to me that you should want Dr. Gus, who has just graduated, and has had no experience, in preference to his father or Dr. Smith."

She said, "Something urges me to have Dr. Gus. I feel that I am going to have a severe and protracted illness and if you don't send for him I won't have any doctor."

Her pleadings prevailed and I was sent for.

I found a typical case of "old-fashioned" typhoid fever as described in the works on Practice of G. B. Wood, Watson and Elliotson. I had seen some cases in St. Joseph's Hospital, Philadelphia, while serving my term as *interne* of that institution. This was all of my experience, but her symptoms were such that I thought if the books were right, I had made a correct diagnosis.

She had sudamina, rose-colored spots and petechiæ, tenderness of the abdomen, and considerable tympanites; her tongue was red around the edges and markedly pointed at the end. In two or three days she passed into a semi-comatose condition, which gradually became more pronounced. She lay in this condition for three weeks. Her eyes open and staring, and until a large portion of the upper conjunctiva was covered with a tough film. She could be aroused from her stupor most of the time and would answer questions when propounded to her. While at the height of her illness, her brother, who was a retired physician, called one afternoon and said to her: "Sister Elizabeth, you are dying. You will not live through the night, and if you have any earthly arrangements to make you had better do so; and send for some of the elders of the church—calling them by name—and have them to pray for you."

She did not heed this suggestion, but told her daughter to send at once for me. This was done, and in a very short time I reached her bedside.

Her looks and words I shall never forget.

She said: "Dr. Gus, Brother Thomas says I am dying; that I

will not live through the night. *You* must not let me die. I am not prepared to die. If I die I will go to hell."

Calling her daughter to her, she said: "Mary Frances, you must not let Dr. Gus leave this room to-night. You sit here by my bedside and if he starts to leave and I am asleep, awake me and I will stop him.

This was a critical and unhappy night for me. I put on as brave a face as I could, though my heart was down in my boots, and said: "Mrs. S., I do not think you are dying. Your symptoms are no worse than when I saw you three hours ago." She said to me, "If you go away to-night, I believe I will die, but if you stay, I believe I will get well."

Her brother being a physician of many years' experience, I thought he knew better than I, and that likely she was dying and I did not know it. At any rate, I threw myself across a bed in the room and in half an hour was sound asleep, her daughter keeping the faithful watch at her bedside, and giving nourishment and stimulants as she had been directed. About day I awoke, everything was as still as death. I arose and crept over to her bedside, and found her sleeping sweetly. Felt her pulse and found she had no fever. Stole away to visit two or three other patients near by. Returned in about two hours, just as she aroused from her slumber. The first thing she said to me when I approached her bedside was: "Dr. Gus, I am better. I told you I wouldn't die if you stayed here all night, and I believe now, I am going to get well."

There was no return of fever, and from that night she continued to improve and did get well.

It is a great thing for a young physician to be thrown upon his own resources.

Never did a patient get more careful and persistent attention from a physician than she did from me. I could not sleep at night for thinking about the case. Would get out of bed and study all the books I found in my own and my father's library, and if I got a new idea would order my horse and visit the patient all hours of night to see if I saw an opportunity to put into practice my new ideas.

It it well to mention that previous to this attack of fever, the patient had suffered from malarial enlargement of the liver and chronic aphthous stomatitis for several years and weighed about 100 pounds.

During this attack the stomatitis and enlargement of the liver both disappeared, and in a year she weighed about 200 pounds and afterwards enjoyed excellent health. It is my experience that if a delicate person recovers from a severe attack of typhoid fever, the tendency is to become robust afterwards. On the contrary, if a robust patient has a severe spell of typhoid fever, they are longer—if ever—in recovering from its effects.

The treatment used with Mrs. S. was at first small does of calomel, until the secretions of the liver were thoroughly aroused; turpentine emulsion, turpentine stupes to abdomen, and a teaspoonful each of kali (effervescing) and sweet spirits nitre, combined, every two, three or four hours in one-half glass of ice water. With this I never failed to produce moisture of the skin, and it had the other desired effect of keeping up an active secretion of the kidneys; in my mind one of the important factors in the treatment of fevers.

Adopting my father's rule, I gave from one to three tablespoonfuls of milk and corn-meal mush every two hours night and day. It was prescribed just as medicine and given just as strictly.

I used milk punch, wine whey, and such other nourishment as the patient could take.

In a word, my object was to nourish and stimulate and thus preserve the vital powers of the patient. To prevent, as far as possible, the ulcerative process of the glands of the intestines, and prevent hemorrhage, if possible. When the temperature was high it was reduced by constant sponging with quinine and whisky (ʒ 1 to O 1). In sweet spirits nitre and citrate potassium (effervescing) we have one of the very best refrigerants in fevers.

We had no thermometers in those days, and the physician had to rely upon the pulse and the touch of the surface of the body as an index of the temperature of his patients, and it is wonderful how reliable this was to the experienced physician of those days.

My patient recovered, became very stout and healthy, and lived a number of years afterwards.

